# Supplementary material for: Patterns of Intron Gain and Loss in Fungi
Source: PLoS Biol. 2004 Nov 30;2(12):e422. doi: 10.1371/journal.pbio.0020422 (PMC532390; doi:10.1371/journal.pbio.0020422)
Supplement: Table S1 — Also available at http://genes.mit.edu/NielsenEtAl/. (4.3 MB ZIP). [file pbio.0020422.st001.zip › NielsenEtAl/html/1280.html]

AN9086.1.NCU05796.1.MG04694.1.FG02649.1


```
 CLUSTAL W (1.82) Multiple Sequence Alignments - Introns Inserted


Sequence 1: NCU05796.1	148 aa
Sequence 2: MG04694.1	189 aa
Sequence 3: FG02649.1	189 aa
Sequence 4: AN9086.1	188 aa
Alignment Length: 190 aa
Number Identitical Residues: 103 aa
Alignment Score (without introns) 5322


MG04694.1 	-MAANKAARVGEE2TRIDKVNAELVTLTYGTIVAQLCKDFDSDYVE~VNKQLDKMGYNIG
NCU05796.1	MSSASKASRLGEE2TRIEKVNAELVVLTYGTIVAQICKDFENDYAE0V------------
FG02649.1 	-MASNKATRLGEE2TRIDKVNAELVTLTYGTIVAQLCKDYESDYAE~VNKQLDKMGYNIG
AN9086.1  	-MSSTKVSRIGEE2NKIDKVNAELVTLTYGTIVAQLCQDYDGNYPE~VNKQLEKMGYNIG
          	  ::.*.:*:*** .:*:*******.*********:*:*::.:* * *... .. . . .

MG04694.1 	LRLIEDYLAKSNTMKRCLNFRETAEMIAK0VGFKIFLNTTPTITNWTGDSKQFSLVFEEN
NCU05796.1	-----------------------------~-GFKIFLNVTPTITNWTGDNKQFSLVFDEN
FG02649.1 	LRLIEDYLAKSNTMKRCANFRETAEMISR0VGFKIFLNITPQVLNWTSENDQFSLVFEEN
AN9086.1  	MRLIEDFLAKSN-AQRCANFRETADMISK0VGFKIFLNITPTVTNWASDNNQFSLIFDEN
          	    ..  :.:.  . . .  .::.  :   ******* ** : **:.:..****:*:**

MG04694.1 	PLADFVELPDDGRAQDELWYSNILCGVLRGALEM~VQMQVEAHFISDVLRGNDTTEMRIS
NCU05796.1	PLADFVELPDDGRAQDQLWFSNILCGVLRGALEM~VQMQVETHFISDVLRGHDTTEMRVT
FG02649.1 	PLADFVELPDDGRAQDQLWYSNILCGVLRGALEM~VQMQVEAHFVSDVLRGNETTEMRVS
AN9086.1  	PLADFVELPDDGRAQDELWFSNILCGILRGALEM0VQMQIEAHFVSDILRGDDTTEMRVS
          	****************:**:******:******* ****:*:**:**:***.:*****::

MG04694.1 	LVRYIDDELPPEDD
NCU05796.1	LVRYIDDELPPEDD
FG02649.1 	LVRYLDDELPPEDD
AN9086.1  	LVRYIEDEVPPESD
          	****::**:***.*
```
